# A priority oriented nutrition education program to improve nutritional and cardiometabolic status in the workplace: a randomized field trial

**DOI:** 10.1186/s12995-020-0252-y

**Published:** 2020-02-13

**Authors:** Bahar Hassani, Reza Amani, Mohammad Hussein Haghighizadeh, Marzieh Araban

**Affiliations:** 1 Department of Nutrition, Ahvaz Jundishapur University of Medical Sciences, Ahvaz, Iran; 2 Department of Health Safety and Environment (HSE), Razi Petrochemical Company, Mahshahr, Iran; 3 Department of Clinical Nutrition, School of Nutrition and Food Science, Food Security Research Center, Isfahan University of Medical Sciences, Isfahan, Iran; 4 Department of Biostatistics and Epidemiology, School of Public Health, Ahvaz Jundishapur University of Medical Sciences, Ahvaz, Iran; 5 Social Determinants of Health Research Center, Ahvaz Jundishapur University of Medical Sciences, Ahvaz, Iran

**Keywords:** Workplace, Nutrition education, Employees, Cardiometabolic markers

## Abstract

**Background:**

Workplace nutrition has been identified as a priority setting that can significantly reduce cardiovascular diseases (CVD) risk factors. This study was conducted as a part of the workplace education program to improve nutritional practices and cardiometabolic status in industrial personnel.

**Methods:**

The present research was a randomized controlled field trial conducted on employees of a regional petrochemical company. The health-related priorities of the program were defined and addressed in the study in which 104 employees with dyslipidemia were randomly divided into two groups of education and control. Data were collected pre- and post-intervention, using valid and reliable multi-session questionnaires on demographic data, nutritional knowledge, and nutritional intake. Anthropometric measures, serum FBS, HbA1C, hs-CRP and homocysteine (Hcy) were assessed in both groups. In the education group, the nutrition program included five educational workshops about healthy nutrition and regular exercise along with educational messages over a 3-month period. The controls did not receive any education during the study.

**Results:**

There were no statistically significant differences between the two groups regarding the baseline variables. The education group significantly improved their nutritional knowledge (*p* < 0.001), dietary intakes (*p* < 0.005), serum FBS (p < 0.001) and Hcy levels (p < 0.001) and anthropometric indices.

**Conclusion:**

Workplace nutrition education programs can improve knowledge and reduce important CVD risk factors.

## Background

Cardiovascular disease (CVD) is a major cause of death worldwide. It will be the most common cause of mortality by the year 2020. Since unhealthy behaviors are major risk factors for CVD, education on healthy lifestyle behaviors is regarded as a health priority [1]. The lifestyle recommendations of the American Heart Association (AHA) [2] promote healthy nutrition and lifestyle practices to decrease major CVD risk factors. Management of these risk factors through modification of lifestyle behaviors can markedly improve the risk of CVD and stroke [3].

Lifestyle management including proper dietary practices aimed at CVD prevention can lead to a lower risk of CVD, improve health status and productivity in the workplace [4]. The AHA has issued guidelines with approaches to promote healthy diets in the workplace including the use of well-balanced meals. These guidelines include consuming fruits and vegetables, whole grains, low-fat dairy products, seafood, lean meats and poultry, salt alternatives, as well as limiting saturated fat, avoiding trans fats, and the provision of nutritional labeling in catering and vending machines [2].

Workplace nutrition education programs can be effective in reducing various risk factors and short-term absenteeism, increasing work efficiency and lowering employees’ healthcare costs [5]. Moreover, nutrition education in the workplace has effectively improved lifestyle habits in terms of diet and physical activity, resulting in lower CVD risk factors [6, 7].

Several reviews on workplace interventions have shown that education programs based on diet, exercise, and lifestyle factors have generally led to improved dietary intakes [8, 9]. Intervention strategies such as from providing health education opportunities to improving nutritional knowledge, attitudes and risk factors of CVD have previously been reported to improve CVD risk factors in employees of a large petrochemical company [10]. Furthermore, as reported in a position statement from the American Heart Association, [11], worksite nutrition interventions had the highest positive effect on health behaviors.

Nutrition education intervention can be more effective when supported by a model or theory, specifically addressing changes in nutrition behavior [10]. The theory of planned behavior (TPB) is an individual-focused health behavior theory used to understand a variety of health behaviors. This theory indicates that individual behavior is affected by four determinants: the individual’s attitude toward behavior, subjective norms, perceived behavioral control and behavioral intention [12].

Petrochemical companies are regarded as pivotal large industries, where improving the workplace conditions can lead to employees’ satisfaction and higher productivity levels [13]. In this randomized controlled field trial, we assessed the effectiveness of a nutrition education program based on TPB model in comparison with routine safety care in petrochemical employees. The primary outcome was the improvement in nutritional behaviors [10]. The secondary outcomes included (i) increase in nutritional knowledge; (ii) improvement in dietary intake, defined as decreased intake of sweets and pastries, soft drinks, junk foods and snacks and increased olive oil consumption; (iii) weight loss and decreased BMI; and (iv) improvement in metabolic CVD risk factors including fasting blood sugar (FBS), hs-CRP and homocysteine (Hcy) levels. According to the authors’ knowledge, no research has assessed the effectiveness of a priority-oriented worksite nutrition program to improve dietary practices and CVD risk factors among the employees of a petrochemical company.

## Materials and methods

### Study design

The present study was a randomized controlled field trial in which all data were collected from June to September 2016. Pre- and post-tests were used to evaluate the participants’ improvement in CVD risk factors including serum lipid profile, fasting blood sugar (FBS), hemoglobin A_1C_ (HbA_1C_), C-reactive protein (hs-CRP), homocysteine (Hcy). Body weight, body mass index (BMI), body fat percentage and dietary intakes were also measured.

Participants were asked to complete the knowledge, TPB and FFQ questionnaires. All participants provided their written consent. The results of TPB components and serum lipid profile are reported elsewhere [10].

### Participants

Participants were male employees of Razi Petrochemical Company in Mahshahr, Khuzestan province, South-West of Iran, located by the Persian Gulf and were selected from four shifts of operational department as the intervention or control group. They had at least 3 years history of employment in the same category while having one blood lipid abnormality. The sample size was determined based on the primary information for serum cholesterol obtained from the study by Allen et al. [14]. Taking the α-value of 0.05,power of 90%, and a possible drop-out of 20%,a sample size of 104 employees with dyslipidemia was required.

In order to prevent the communication and distribution of information among participants, participants working in 4 shifts were selected by random number table method. Due to the almost equal distribution of the samples selected in each shift, two shifts (A&B) as the intervention group and two shifts (C&D) as the control group were randomly selected. Participants were then randomly assigned as 52 subjects in the education and control groups using random number generation. Randomization was individually achieved using sealed envelopes by the help of a research assistant.

The control group received routine medical care. The education group received routine medical care plus the nutrition education program. Inclusion criteria were male personnel that were shift workers in four shifts, working at least in 72-h shift cycles (12 days) including 3 shifts from morning, evening and night shift staff. Individuals who were not willing to participate in educational classes, those using any medications and alcohol, regular smokers as well as those having a history of disease such as hyperthyroidism and hepatorenal dysfunction were excluded from the study.

Demographic features such as age, weight, and height, body mass index (BMI), medical history, and alcohol consumption were obtained through questionnaire and from medical records. Written informed consent was obtained from all employees. The participants’ age ranged from 30 to 60 years and they were similar in terms of job category, education and income levels. Finally, 49 in the intervention and 43 in the control group met the full criteria to enter the study.

### Objective measures

All participants were evaluated at baseline and following three months intervention. Each test session lasted approximately one hour and included completion of questionnaires, anthropometric measures and blood sampling. Height was measured to the nearest mm without shoes. Body weight and percent body fat were measured using a bioimpedance analysis device (OMRON BF-511; Japan), while body frame and the participant’s age, height and gender were entered. Body weight was measured while the participant had minimal clothing on with no shoes. BMI was calculated as body weight (kg) divided by squared height (m^2^).

### Blood sampling and biochemical assays

Fasting blood samples were obtained pre- and post- intervention. All subjects were asked not to eat food for 12 to 14 h overnight. Five mL fasting blood samples were taken to evaluate the blood biochemical parameters [15]. Serum hs-CRP, FBS and Hcy concentrations were measured by enzymatic methods using ELISA method and HbA_1_c was measured by Immunoturbidimetric using Pars Azmoon Kits (Karaj, Iran). All equipment were routinely calibrated at the beginning of each workday using the standard protocol provided by the manufacturers.

### Questionnaires

Each questionnaire was completed twice, one week prior to each test and 3 months following the educational intervention (Additional file 1).

#### (i) The demographic and anthropometric questionnaire

This questionnaire measured variables such as age (y), work experience (y), number of children, marital status, educational level, monthly income, body weight, BMI and body fat percentage. BMI ranging from 18.5 to 24.9 was considered as normal, under 18.5 was regarded as underweight, equal or over 25 as overweight and over 30 as obese [16].

#### (ii) Nutritional knowledge

Nutritional knowledge was characterized as the self-perception of the importance to eat balanced meals [17]. A questionnaire was developed for all employees in the educational program consisting of 20 standardized and validated knowledge questions regarding nutritional behaviors, healthy diet, cardiovascular risk factors, weight management and exercise [10]. Answers were scored based on the ranking scale, from 5 (true answer) to 0 (false answers). The knowledge scores ranged from 0 to 100.

#### (iii) Dietary intake

To measure the effectiveness of employees’ nutritional behaviors, we applied a short validated food frequency questionnaire (FFQ) including 28 selected main food groups [18]. The validity of the questionnaire was measured by both content and face validity by seeking the opinion of an expert panel. To do so, comments of five experienced professors were obtained according to Waltz and Bussel recommendation [19] and included in the questionnaire. To assess the face validity, the questionnaire was given to 30 employees (homogenous and non-participants). Further comments, questions and notes pointed out by this group were considered. The questionnaire reliability was measured through the Cronbach’s alpha method for knowledge and internal consistency of 0.75 was obtained. Cronbach’s alpha statistic is widely used in the social sciences, nursing, and other disciplines to measure internal consistency. We found an acceptable level of 0.8 as Cronbach’s alpha for TPB constructs.

### Educational intervention

Before designing the educational plan, five year records of CVD risk factors were obtained from the health, safety and environment (HSE) department. Using these data, the items with the highest health priority and prevalence in the staff were included in the educational program [20]. The educational intervention was performed in 3 months. Five training sessions were aimed at avoiding the intake of trans-fats, using less saturated fats and simple carbohydrates, increasing the consumption of fruits/vegetables and whole grains while highlighting the importance of breakfast and healthy snacks through educational classes at work. Educational content was delivered through lectures, question/answer sessions and group discussion. At the end of each session, a package of healthy snacks was distributed.

The details of individual training sessions were as follows. The first and second sessions (1st week and 3rd week) included CVD and its signs, complications, diagnosis, risk factors, obesity and weight management. This included the provision of educational materials such as booklets to the intervention group and their families. The third and fourth sessions (5th week and 7th week) underscored the role of healthy diet and physical activity in reducing the risk factors of CVD and the benefits of following proper dietary recommendations through question and answer sessions. The last session (9th week) was conducted for the family members of intervention group, in which the importance of family in preparing, facilitating, and providing suitable foods as well as the role of physical activity programs in improving CVD risk. To enhance nutritional knowledge in the classroom, we provided a PowerPoint presentation, and a booklet compiled by the research team. Validated text messages were also prepared by project team and three messages were sent every week automatically.

Question and answer sessions and telephone follow-ups were performed at 4th and 8th week of the intervention to reinforce the educational contents, the role of the family and also to provide answers to any raised questions. Finally, the questionnaires were completed and blood samples were collected at week 12. To observe the research ethics code, the contents of the program were provided to the control group over two sessions at the end of study. The study protocol was approved by the Medical Ethics Committee at the Ahvaz Jundishapur University of Medical Sciences, Ahvaz, Iran.

### Statistical analysis

Statistical analysis was performed using SPSS version 20. Demographic variables were compared between the groups using the Chi-square test. Knowledge, anthropometric factors, nutritional intake, and blood parameters were analyzed within and between the groups using paired t-test and independent t-test, respectively. ANCOVA test was also applied to control the effects of possible confounders.

Statistical significance was determined at *p*-value < 0.05 level. All data were normally distributed. There were no missing values in the dataset [21].

## Results

**Fig. 1:**
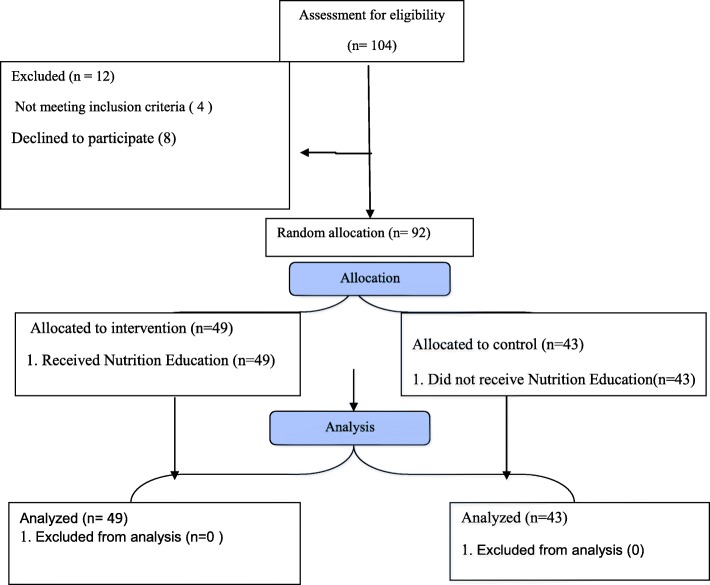
Flow diagram of the study

A total of 92 participants finished the study. Three persons in the education group and nine persons in the control group were excluded due to personal reasons (Fig. 1). Table 1 indicates the demographic criteria of the subjects.

**Table 1:** Demographic Variables of the Study Participants

Variables	Control	Education	*P* value^a^
Age (y)	43.18±8.75	42.3±9.13	0.64
Work experience (y)	19.55±8.67	18.05±9.33	0.58
Number of children	1.69±1.08	1.61±1.01	0.69
Marital Status			
Married	41 (95.3%)	45 (91.8%)	0.681
Single	2 (4.7%)	4 (8.2%)	
Education Level			
Diploma	10 (23.3%)	13 (26.5%)	0.63
Associate′s Degree	2 (4.7%)	3 (6.1%)	
B.A	24 (55.8%)	26 (53.1%)	
M.S and higher degrees	7 (16.3%)	7 (14.3%)	
Monthly Income (million Rls)			
Less than 30	6 (14%)	4 (8.2%)	0.66
30-50	30 (69.8%)	36 (73.5%)	
Above 50	7 (16.2%)	9 (18.4%)	

All tests are conducted at significant level of 0.05

^a^Based on independent t-test

No significant differences were seen in terms of demographic and nutritional knowledge variables. However, after 3 months of educational intervention, the intervention group showed above 50% improvement in their nutritional knowledge (*P* < 0.001, Table 2).

**Table 2:** Knowledge of control and education groups

Variables	Control (*n* = 43)	Education (*n* = 48)	*P* value^a^
Knowledge			
Baseline	45.81±20.84	50.72±21.87	0.330
Follow-up	47.81±17.48	75.29±17.07	<0.001
Change	2.00±21.00	24.56±25.64	0.000
*P* value^b^	0.536	<0.001	

All tests are conducted at significant level of 0.05

^a^Based on independent t-test

^b^Based on paired t-test

Body weight and BMI were lowered by 0.7 kg and 0.4 kg/m2 respectively in the intervention group (*p* < 0.05, Table 3).

**Table 3:** Anthropometrics variables of the study groups

Variables	Control (*n* = 43)	Education (*n* = 48)	*P* value^a^
Weight (kg)			
Baseline	86.82±12.54	86.83±10.81	0.996
Follow-up	87.29±13.33	85.60±10.39	0.501
Change	-0.46±2.41	-1.35±1.93	0.000
*P* value^b^	0.214	<0.001	
BMI			
Baseline	28.46±3.57	28.73±3.16	0.713
Follow-up	28.58±3.71	28.25±3.02	0.641
Change	0.11±0.96	-0.48±1.06	0.006
*P* value^b^	0.426	<0.005	
Body Fat* (%)			
Baseline	24.05±5.60	26.59±5.88	0.039
Follow-up	25.32±5.60	26.37±4.47	0.136
Change	1.27±5.6	-0.22±3.72	0.136
*P* value^b^	0.147	0.683	

*Comparison based on baseline values of body fat percent. ANCOVA test was also applied to control the effects of possible confounders

^a^Based on independent t-Test

^b^Based on paired t-test

There were no significant differences between the groups regarding the intake of sweets, soft drinks, cake and cookie, and snacks. However, the intervention group revealed significant declines in all mentioned food items from the baseline (p < 0.05, Table 4).

**Table 4:** Dietary intakes of the control and education group, pre- and post-education

Food		Before		*P* value^b^	After		*P* value^a^
		Education	Control		Education	Control	
Sweets and pastries	2 or more per day	3 (6.97)	3 (6.12)	0.919	3 (6.97)	1 (2)	<0.005
	Once a day	2 (4.65)	2 (4.80)		3 (6.97)	1 (2)	
	Several time per week	9 (20.93)	15 (30.61)		10 (23.2)	10 (20.4)	
	1-4 times per month	12 (7.90)	10 (20.40)		14 (5.32)	19 (44.1)	
Control							0.05
Education							0.823
Soft drinks	2 or more per day	4 (9.30)	2 (4.08)	0.980	2 (4.65)	0	<0.005
	Once a day	9 (20.93)	5 (10.20)		2 (4.65)	0	
	Several time per week	12 (7.90)	17 (34.69)		16 (7.20)	13 (26.53)	
	1-4 times per month	13 (30.23)	12 (24.48)		12 (7.90)	18 (36.73)	
Control							0.005
Education							0.662
Cake and cookie snacks	2 or more per day	3 (7)	4 (8.16)	0.259	5 (8.3)	10 (17.6)	0.147
	Once a day	4 (9.3)	11 (22.44)		7 (16.27)	5 (10.2)	
	Several time per week	18 (41.7)	14 (28.6)		12 (20)	35 (61.4)	
	1-4 times per month	10 (23.25)	15 (30.6)		25 (41.7)	10 (17.6)	
Control							0.021
Education							0.575
Olive oil	2 or more per day	4 (9.30)	1 (2)	0.018	4 (9.30)	3 (6.12)	<0.005
	Once a day	12 (27.9)	7 (14.3)		7 (16.27)	9 (18.36)	
	Several time per week	10 (23.2)	14 (28.6)		19 (44)	18 (36.73)	
	1-4 times per month	9 (20.93)	17 (34.7)		8 (18.60)	10 (20.40)	
Control							0.150
Education							0.209
Sugars	2 or more per day	13 (30.2)	23 (46.93)	0.026	13 (30.2)	13 (26.53)	<0.005
	Once a day	6 (13.95)	8 (16.32)		8 (18.60)	13 (26.53)	
	Several time per week	12 (27.9)	9 (18.36)		7 (16.27)	9 (18.36)	
	1-4 times per month	4 (9.30)	7 (14.3)		5 (11.62)	7 (14.23)	
Control							0.024
Education							0.823

All tests were conducted at a significant level of 5%

^a^Based on independent t-Test

^b^Based on paired t-test

FBS and HbA1c levels were improved in the education group (p < 0.05, Table 5).

**Table 5:** Serum levels of serum biochemical parameters of the control and education groups

Variables	Control (*n* = 43)	Education (*n* = 48)	*P* value^a^
FBS (mg/dL)			
Baseline	82.09±17.15	80.27±10.48	0.528
Follow-up	98.97±20.30	83.68±13.89	<0.001
Change	16.88±21.92	3.41±13.07	0.001
*P* value^b^	<0.001	0.08	
HbA1C(mmol/L)			
Baseline	4.50±0.54	4.58±0.56	0.489
Follow-up	4.75±0.48	4.60±0.49	0.164
Change	0.24±0.69	0.20±0.43	0.067
*P* value^b^	0.026	0.743	
hs-CRP (mg/dL)			
Baseline	2.08±2.82	2.09±2.94	0.738
Follow-up	2.30±2.43	3.36±10.57	0.525
Change	0.22±1.38	1.26±10.57	0.524
*P* value^b^	0.299	0.412	
Hcy (μmol/L)			
Baseline	23.54±9.94	23.58±10.56	0.838
Follow-up	29.44±9.41	21.71±7.61	<0.001
Change	5.90±6.98	2.1±5.24	0.001
*P* value^b^	<0.001	0.006	

All tests are conducted at a significant level of 5%

^a^Based on independent t-Test

^b^Based on paired t-test

Serum levels of hs-CRP showed no significant changes while Hcy levels were reduced to 1.87 μmol/L (*P* value < 0.05) in the intervention group. Intra-group changes in serum Hcy levels were also statistically significant (Table 5).

## Discussion

Cardiovascular diseases are among the most common causes of mortality globally, accounting for more than half of the mortality worldwide. Despite the remarkable progress in health care and treatment, there has been a significant increase in lifestyle-related CVD among general employees [22, 23]. In recent years, there has been increasing interest in the concept of workplace health promotion and wellbeing as a strategy to reduce the burden of CVD [24, 25].

Results of the present study highlight the importance of applying educational programs to control the risk factors in industrial employees. This outcome could be due to the lack of knowledge about the CVD risk factors and its prevention methods. Indeed, employee nutrition-related knowledge was increased significantly and other key determinants such as body weight, BMI, dietary intakes and biochemical parameters were also improved. Effectiveness of the educational program on the improvement of CVD in dyslipidemia employees was also observed [10]. Encouraging lifestyle modifications can delay or prevent the onset of CVD by reducing the main riskfactors (Table 2). Our findings suggest that improved employee knowledge and their dietary pattern may improve cardiovascular disease risk, and hence, provide evidence regarding the importance of tailored nutrition education programs in CVD prevention.

Encouraging lifestyle modifications can delay or prevent the onset of CVD by reducing the main risk factors (Table 2). Lifestyle interventions focusing on dietary pattern correction should be promoted in all worksites especially in environments with higher risks [26].

Effectiveness of the educational program on the improvement of CVD in dyslipidemia employees was also observed [10]. This outcome could be due to the lack of knowledge about the CVD risk factors and its prevention methods. The results indicate that there is inadequate knowledge and poor employees performance in avoiding risk factors and predisposing behaviors of heart disease, while in many cases, by improving risky behaviors such as smoking, inappropriate diet, sedentary lifestyle and alcohol consumption, high incidence of CVDs and other non-communicable diseases can be prevented [27].

As stated by Chivanidze et al., knowledge about proper nutrition, nutritive foods and healthy eating practices can improve the health of the society and country as a whole [28]. Prominent scientific communities have recommended primary prevention, such as raising knowledge about CVDs and their risk factors, as the most essential and cost-effective approach to control the CVD [29]. In addition, nutritional knowledge has a profound influence on food choices, nutritional habits and nutrient intakes [30]. Theoretical models suggest that individual knowledge of food is a key determinant of food choices [31]. Therefore, self-perception of the importance of balanced meals (i.e. nutritional knowledge) can be viewed as an important factor that can influence dietary choices and nutritional intake [32].

Nutritional knowledge was improved in the intervention group (Table 2), which is in agreement with the results of other research [33]. Our findings are also confirmed by the study of Graney et al. (2016) that applied a workplace dietary intervention to increase nutrition knowledge, health status and to improve dietary intake among the employees [34] In addition, Thomason et al. (2018) applied a 12-week online nutritional education program that was designed and taught by registered nutritionists in the workplace to enhance healthy diet among employees [35] However, Sun et al. (2016) reported no statistically significant changes in nutritional knowledge following a similar intervention [36]. Their results indicated that changing knowledge through educational intervention is not always a simple goal to achieve. Our intervention group also consumed fewer sweets, soft drinks (such as soda), cake and cookies, snacks such as French fries, chips and sugars, which are in accordance with other studies [37].

Providing accessible materials, planning sessions with applicable points and also involving families are regarded as the possible reasons for improving the participants’ knowledge [10]. Research has shown that overweight/obese employees exert significant healthcare burdens for employers [38]. A significant decrease in body weight and BMI was reported in this study, which is in line with Braham et al. [39]. However, the change in body fat was unlikely to be of clinical importance. Decreasing the average body weight and BMI in the education group may imply the effectiveness of education program in reducing CVD risk.

The changes in serum FBS levels were within the normal range (Table 5); however, the increment in the control group was significant. Salinardi et al. reported that workplace educational intervention could improve the serum levels of FBS in the intervention group. They showed that a higher intake of dietary fiber and foods with lower glycemic index as well as controlling body weight and energy intake can lead to improved glycemia status [40].

There is an association between diet quality and inflammatory markers. Studies showed that increasing hs-CRP serum levels is associated with elevated risk of CVD in different population around the world [41]. Mortality from CVDs is 2-fold higher when hs-CRP is above 3 mg/L. This concentration is considered as high risk by the AHA and CDC, compared to hs-CRP levels below 1 mg/L [41].

In our study, the serum levels of hs-CRP showed no significant changes in the two group (Table 5), which is in line with Barham et al. [39]. The educational model also improved the serum levels of Hcy that is regarded as an important CVD risk factor described in previous studies [42, 43]. The reasons for a significant decrease in the level of Hcy could be due to increased knowledge and improving nutritional behavior. As stated by Dawkins et al., higher intake of folate through increased consumption of salads and raw vegetables, reduced consumption of red meats and more importantly improving nutritional behaviors could be regarded as the reasons for lowered Hcy level [42, 43].

## Limitations

Among the limitations of our study, the resources prevented us from extending the study to more than three months. Moreover, the exact comparison between our findings and those of previous works was not possible due to the scarcity of similar designs or absence of similar findings in the same conditions. Our study was conducted in male employees only, and therefore, cannot necessarily be generalized to female staff. We used FFQ to assess dietary behaviors that could be subject to recall bias. A nutritionist (BH) who was educated by a health education specialist (MA) conducted the educational session. There was no incentive for the participants and we did not evaluate the effect of the intervention on participants’ family.

One of the strengths of our study was preparing a practical guidebook in which all aspects of improving dietary practice were simply described in similar workplace with the same conditions. Another strength was that the educational program was tailored based on the HSE requirements and health priorities that were reported through annual checkups. The inclusion of additional strategies to improve adherence and uptake of the intervention such as the provision of accessible materials, planning sessions with applicable points and involving families are also strengths of this study [10]. It was also strength that all participants completed the study course. We conducted all educational sessions on schedule and the acceptance rate was 100%.

## Conclusion

The findings showed the effectiveness of worksite nutritional program in a main oil industry and highlighted the importance of nutrition education interventions to improve awareness, dietary practice, anthropometric measurements as well as important cardiometabolic risk factors. Implementing work-place nutrition education programs using an appropriate educational model based on health-related priorities can be applicable in similar industries.

## Additional file

**Additional file 1.** Reaserch questionnaire.

## Data Availability

The dataset generated and analyzed in this study are not publicly available due to considerations of data protection but are available from the corresponding author (RA) on reasonable request.

## Abbreviations

AHA: American Heart Association
BMI: Body Mass Index
BMI: Body Mass Index
BMI: Body Mass Index
CVD: Cardiovascular Disease
FBS: Fasting Blood Sugar
FFQ: Food Frequency Questionnaire
HbA1C: Hemoglobin A1C
Hcy: Homocysteine
hs-CRP: hs-C-reactive protein
HSE: Health Safety Environment
TPB: Theory of Planned Behavior
