# Supplementary material for: A priority oriented nutrition education program to improve nutritional and cardiometabolic status in the workplace: a randomized field trial
Source: J Occup Med Toxicol. 2020 Feb 13;15:2. doi: 10.1186/s12995-020-0252-y (PMC7020354; doi:10.1186/s12995-020-0252-y)
Supplement: Supplementary file 1 — Additional file 1. Reaserch questionnaire. [file 12995_2020_252_MOESM1_ESM.docx]

**Research Questionnaire**

**Appendix A**

**Demographic characteristics**

1. Sure name, Family name:
2. Phone Number:
3. Sex:
4. Age: ………… years old
5. Educational Attainment level: □ Diploma □ Associate′s Degree □ B.A □ M.S and higher degrees
6. Monthly income level(million Rls): □ Less than 30 □ 30-50 □ Above 50
7. Marital status: □ Single □ Married
8. Employment status: □ Official □ Contractual
9. Work experiences: ………… years old
10. Number of children: …………
11. How do you evaluate your monthly income: □ Low □ Medium □ High □ Very High □ Perfect

**Appendix B**

**⮚ Knowledge questions:**

1. Removing what kind of daily meal due to obesity?
   1. Breakfast
   2. Lunch
   3. Dinner
   4. Lunch and Dinner
   5. I do not know All of the above
2. What is the normal range of BMI in adult people≤?
   1. ≤ 18.5
   2. 25-29.9
   3. 18.5-24.9
   4. ≥30
   5. I do not know
3. What is a minimum serving of fruits and vegetables in days?
   1. 1-2 servings in a day
   2. 2-3 servings in a day
   3. 3-5 servings in a day
   4. 2.5-4 servings in a day
   5. I do not know
4. Which types of food can prevent dyslipidemia?
   1. Whole grain, vegetables and fruits
   2. Refined grain, saturated fat and soft drinks
   3. Whole grain and trans fat
   4. Refined grain and low fat dairy
   5. I do not know
5. How much recommended measure of waste circumference (WC) in male and female for preventing cardiovascular disease?
   1. In male ≤102 cm and in female ≤88
   2. In male and female ≥95
   3. In male 100 cm and In female 90 cm
   4. In male and female 90 cm
   5. I do not know
6. Which type of below item can be a healthy snack in home or workplace?
   1. Snack is including: high fiber, high trans-fat and low sugar
   2. Snack is including high fiber, high trans-fat and low sugar
   3. Snack is including low fiber, high trans-fat and low sugar
   4. Snack is including low fiber, high trans-fat and high sugar
   5. I do not know
7. What is the best way of loss weight in obese and overweight people?
   1. Only increasing physical activity
   2. Decreasing calorie intake in dinner
   3. Decreasing calorie intake in all day plus increasing physical activity
   4. Removing breakfast plus increasing physical activity
   5. I do not know
8. What is minimum physical activity to gain healthy weigh in a day?
   1. 30 minutes in 5 days of week
   2. 15 minutes in 5 days of week
   3. 30 minutes in 3 days of week
   4. 10minutes in 3 days of week
   5. I do not know
9. In Iran, what is the first reason of death?
   1. Cardiovascular disease
   2. Censer
   3. Infectious disease
   4. Diabetes
   5. I do not know

10. Which one of below nutrient in fish can prevent cardiovascular disease?

a. saturated fatty acid

b. Omega 3 fatty acid

c. Omega 6 fatty acid

d. Protein

e. I do not know

11. What is the below food including trans-fat:

a. Margarine and biscuit

b. vegetables oil

c. chicken and fish

d. Notes and seeds

e. I do not know

12. Which one of below food Sodium most of intake Na of food

a. Vegetables and food

b. Dairy

c. Processed food

d. Oils

c. I do not know

13. In banana, melon, orange, watermelon, tomato, broccoli and celery there is an effective mineral that can prevent hypertension. It is…

a. Sodium

b. Cupper

c. Iron

d. Potassium

e. I do not know

14. Which one of lipid parameters is affected by usage of olive oil?

a. HDL.C

b. LDL.C

c. Cholesterol

d. Triglyceride

e. I do not know

15. About smoking, what is most important as risk factor of cardio vascular disease?

a. Time of smoking

b. Amount of cigarette

c. Variety of cigarette

d. None of above

e. I do not know

16. Which one of below oils including Oleic acid that can prevent cardiovascular disease?

A. Olive, Canola

b. Sunflower, Corn

c. Soy, Palm

d. Coconut0, Olive

e. I do not know

17. What is the best cooking way to prevent dyslipidemia?

a. Grill

b. Steam

c. Frying

c. tandoori

e. I do not know

18. Which one of below food has more effect for increasing of blood cholesterol?

a. Meat

b. Shrimp

c. Egg

d. Vegetables oil

e. I do not know

19. What is the best diet for losing weight and keeping healthy weight?

a. Low calorie diet

b. Low fat diet

c. Low carbohydrate diet

d. High protein diet

e. I do not know

20. What is the most harmful diet that affect on lipid parameters?

a. Unsaturated fat

b. Trans fat

c. Dietary cholesterol

d. Saturated fat and trans fat

e. I do not know

Appendix C

**Theory of planned behavior**

Nutrition approaches for management dyslipidemia including:

1. Reducing fatty acid sources intake intake (hydrogenated oil, animal fat, meat, high fat dairy, ...)
2. Reducing trans fatty acid (vegetables oil, margarine, biscuit, sweet and pastry and all the food that make hydrogenated oil)
3. Increasing sources of fiber (vegetables, fruits, cereal, whole grain,…)
4. Increasing physical activity ( 30-45 walking in the most days of week)
5. Reducing calorie intake and body weight management.

**Appendix B**

**⮚ Attitude questions**

| To me, due to my job, management of calorie intake and controlling body weight is… | Very hard | Hard | Neither easy nor hard | Easy | Very easy |
| --- | --- | --- | --- | --- | --- |
| To me, increasing usage of fruits and vegetables is ……… | Very possible | Possible | Neither possible nor impossible | Impossible | Very impossible |
| To me, usage homemade food prefer than junks food ……… | Very agree | Agree | Neither agree nor disagree | Disagree | Very disagree |
| To me, 30-45 minutes fast walking in most days of week cause of increasing physical and mental situation ……… | Very possible | Possible | Neither possible nor impossible | Impossible | Very impossible |
| To me, 30-45 minutes fast walking in most days of week cause of increasing physical and mental …….. | Right | Neither right nor wrong | Wrong | Very wrong | Completely wrong |
| To me, 30-45 minutes fast walking in most days of week ….. | Very enjoyable | Enjoyable | Neither enjoyable nor agonizing | Agonizing | Very agonizing |
| To me, usage of olive oil, canola oil, sesame oil and vegetables oil prefer than hydrogenated oil. | Very practicable | Practicable | Neither practicable nor impracticable | Impracticable | Very  impracticable |
| To me, taste and smell hydrogenated oils prefer than olive oil, canola oil, sesame oil and other vegetable oils. | Highly agree | Agree | Neither agree nor disagree | Disagree | Highly disagree |
| I like Fast food, sausages and ham for their taste. | Highly agree | Agree | Neither agree nor disagree | Disagree | Highly disagree |
| Usages of healthy fruits and vegetables due to I will do my work better. | Highly agree | Agree | Neither agree nor disagree | Disagree | Highly disagree |
| When I have healthy food including data, raisin and nuts, I feel more power. | Highly disagree | Disagree | Neither agree nor disagree | Agree | Highly agree |

**Appendix D**

**⮚ Subjective norms questions**

| 1. My family members think I should do more physical activity | Highly agree | Agree | Neither agree nor disagree | Disagree | Highly disagree |
| --- | --- | --- | --- | --- | --- |
| 2 My colleagues think I should do more physical activity. | Highly agree | Agree | Neither agree nor disagree | Disagree | Highly disagree |
| 3. My family members want me to fallow healthy diet. | Highly agree | Agree | Neither agree nor disagree | Disagree | Highly disagree |
| 4. My colleagues want me to fallow healthy diet. | Highly agree | Agree | Neither agree nor disagree | Disagree | Highly disagree |
| 3. It is expected of me to fallow healthy nutritional behaviors. | Highly agree | Agree | Neither agree nor disagree | Disagree | Highly disagree |
| 4. I feel under pressure by workplace to usage of healthy food. | Highly agree | Agree | Neither agree nor disagree | Disagree | Highly disagree |

**Appendix E**

**⮚ Perceived behavioral control questions**

| 1. Physical activity and doing sport is not in my ability. | Highly agree | Agree | Neither agree nor disagree | Disagree | Highly disagree |
| --- | --- | --- | --- | --- | --- |
| 2. Calorie intake from food at workplace is in my control. | Highly agree | Agree | Neither agree nor disagree | Disagree | Highly disagree |
| 3. Reducing unhealthy food including hydrogenated oil, frying oil, margarine, meat and… is easy for me at workplace. | Highly agree | Agree | Neither agree nor disagree | Disagree | Highly disagree |
| 4. Reducing unhealthy food including sugar, cake, biscuit, soft drink and… is easy for me at workplace. | Highly agree | Agree | Neither agree nor disagree | Disagree | Highly disagree |
| 5. I sure if I want to have enough amount of healthy foods includes vegetables, fruits, whole grains at workplace, I can. | Highly agree | Agree | Neither agree nor disagree | Disagree | Highly disagree |
| 6. I able to have healthy snack including fruits, vegetables and low fat dairy at workplace instead of junks food. | Highly agree | Agree | Neither agree nor disagree | Disagree | Highly disagree |
| Acting dyslipidemia approaches is in my control. | Highly agree | Agree | Neither agree nor disagree | Disagree | Highly disagree |

**Appendix F**

**⮚ Intention**

| 1. I am going to during future three month I will get recommended healthy weight. | Highly agree | Agree | Neither agree nor disagree | Disagree | Highly disagree |
| --- | --- | --- | --- | --- | --- |
| 2. I am going to during future three month I will do sport in daily routine. | Highly agree | Agree | Neither agree nor disagree | Disagree | Highly disagree |
| 3. I am expecting myself, to increase eating of fruits and vegetables during future three month; even these foods will be expensive. | Highly agree | Agree | Neither agree nor disagree | Disagree | Highly disagree |
| 4. I am expecting myself, to increase eating of fruits and vegetables during future three month; even I will suppose some problems at workplace. | Highly agree | Agree | Neither agree nor disagree | Disagree | Highly disagree |
| 5. I am expecting myself, to decrease eating of hydrogenated oils, margarine, frying foods and meat during future three month; even I will suppose some problems at home. | Highly agree | Agree | Neither agree nor disagree | Disagree | Highly disagree |
| 6. I am expecting myself, to decrease eating of hydrogenated oils, margarine, frying foods and meat during future three month; even I will suppose some problems at workplace. | Highly agree | Agree | Neither agree nor disagree | Disagree | Highly disagree |
| 7. I am expecting myself, to eat of steamed and grilled foods instead of fried food in my diet during future three month. | Highly agree | Agree | Neither agree nor disagree | Disagree | Highly disagree |
| 8. I am going to do sport during future three month; even I will be so busy. | Highly agree | Agree | Neither agree nor disagree | Disagree | Highly disagree |
| 9. I am expecting myself, to alternate using of unhydrognated oil including olive oil, canola oil, … instead of hydrogenated oils during future three month; even I will not like their taste. | Highly agree | Agree | Neither agree nor disagree | Disagree | Highly disagree |

**Appendix F**

**⮚ Nutritional Behaviors**

Dear Colleague, below questions are asking you about your last three months’ **Nutritional Behaviors**, please answer to the questions carefully:

| Please specify the most convenience reply by X | Always | Frequently | Sometimes | Rarely | Any time |
| --- | --- | --- | --- | --- | --- |
| 1. How often have you consumed fruit and vegetable 3 to 5 times per day? |  |  |  |  |  |
| 1. How often have you had walking body activity for 30 minutes per day? |  |  |  |  |  |
| 1. How often have you had fish per day? |  |  |  |  |  |
| 1. How often have you consumed foods consisting Anti- Oxidant including red and violet fruits such as berry, tomato, carrot, onion,… per day? |  |  |  |  |  |
| 1. How often have you consumed drinks consisting Anti- Oxidant including green tea, black tea and coffee per day? |  |  |  |  |  |
| 1. How often have you increased meals and decrease portion of food due to lose weight per day? |  |  |  |  |  |
| 1. How often have you consumed low fat dairy instead of high fat dairy per day? |  |  |  |  |  |
| 1. How often have you consumed healthy snacks including fruits, vegetables and nuts instead of junk foods per day? |  |  |  |  |  |
| 1. How often have you consumed healthy drink including water and instead of soft drink per day? |  |  |  |  |  |
| 1. How often have you consumed vegetables proteins instead of meat per day? |  |  |  |  |  |
| 1. How often have you consumed food consisting high fiber including cereals, beans and whole grains per day? |  |  |  |  |  |
| 1. How often have you consumed planet butter (margarine) / Solid planet oils (Hydrogenated), cake and biscuit per day? |  |  |  |  |  |
| 1. How often have you consumed fried foods per day? |  |  |  |  |  |
| 1. How often have you consumed Sugar and per day? |  |  |  |  |  |
| 1. How often have you consumed red meat? |  |  |  |  |  |
| 1. How often have you checked blood lipids? |  |  |  |  |  |
| 1. How often have you measured your body weight? |  |  |  |  |  |
| 1. How often have you measured your body fat percent? |  |  |  |  |  |
| 1. How often have you measured your waist waist circumference? |  |  |  |  |  |
| 1. How often have you read about cardiovascular disease? |  |  |  |  |  |

**Appendix F**

**⮚ Food frequency questionaries’**

Specify for each below listed foods in last 6 months, how often have you consumed average?

| Cons. Freq.  Foods | Two ( or more) times in a day | Ones in a day | Few times in a week | One to four times in a month | Ones in each 2 till 3 months | At all |
| --- | --- | --- | --- | --- | --- | --- |
| Cake and sweets |  |  |  |  |  |  |
| Fast Foods (Sandwiches, Pizza, Snakes ….) |  |  |  |  |  |  |
| Junk food (Chips) |  |  |  |  |  |  |
| Butter and Margarine |  |  |  |  |  |  |
| Fresh Fruits / Natural juices |  |  |  |  |  |  |
| Salad/ Raw vegetables |  |  |  |  |  |  |
| Cooked vegetables |  |  |  |  |  |  |
| Drinks ( Cola , Beer , Sweets , synthesis juices, soft drink) |  |  |  |  |  |  |
| Egg |  |  |  |  |  |  |
| Meat |  |  |  |  |  |  |
| Visceral Meats ( liver, heart, Kaleh pacheh) |  |  |  |  |  |  |
| Chicken |  |  |  |  |  |  |
| Fish / Shrimp |  |  |  |  |  |  |
| Low fat dairy(milk ,yogurt , cheese) |  |  |  |  |  |  |
| High fat dairy( milk , yogurt , cheese) |  |  |  |  |  |  |
| Bread was made with whole grains (Barbary, Sangak) |  |  |  |  |  |  |
| Refined cereals( Lavash, Beget, Pasta, Biscuit) |  |  |  |  |  |  |
| Potato |  |  |  |  |  |  |
| Nuts (Sour, Raw) |  |  |  |  |  |  |
| Sweets (confectionary, chocolate, pastry) |  |  |  |  |  |  |
| Sugar |  |  |  |  |  |  |
| Sours |  |  |  |  |  |  |
| Salt (Salt , sour cucumber) |  |  |  |  |  |  |
| Ice-cream |  |  |  |  |  |  |
| Coffee and Tea |  |  |  |  |  |  |
| Hydrogenated Oils (Solid) |  |  |  |  |  |  |
| Un hydrogenated Oils (Liquid) |  |  |  |  |  |  |
| Olive Oil |  |  |  |  |  |  |
